# Methodology for human-induced pluripotent stem cell–derived excitatory and inhibitory neuron coculture with astrocytes for Alzheimer’s disease modelling

**DOI:** 10.1093/braincomms/fcag135

**Published:** 2026-05-11

**Authors:** Jialin Li, Lucy E Brown, Naiomi Rambarack, Andi Chan, Priya Prakash, Charles Arber, Selina Wray, Afia B Ali

**Affiliations:** Department of Pharmacology, UCL School of Pharmacy, London WC1N 1AX, United Kingdom; Department of Pharmacology, UCL School of Pharmacy, London WC1N 1AX, United Kingdom; Department of Pharmacology, UCL School of Pharmacy, London WC1N 1AX, United Kingdom; Department of Pharmacology, UCL School of Pharmacy, London WC1N 1AX, United Kingdom; Department of Pharmacology, UCL School of Pharmacy, London WC1N 1AX, United Kingdom; Department of Neurodegenerative Disease, UCL Queen Square Institute of Neurology, London WC1N 1PJ, United Kingdom; Department of Neurodegenerative Disease, UCL Queen Square Institute of Neurology, London WC1N 1PJ, United Kingdom; Department of Pharmacology, UCL School of Pharmacy, London WC1N 1AX, United Kingdom

**Keywords:** Alzheimer's disease, induced pluripotent stem cell, electrophysiology, interneurons, astrocytes

## Abstract

Alzheimer’s disease symptoms include gradual cognitive decline and memory loss that is correlated with progressive loss of neuronal connections due to an imbalance of excitatory and inhibitory synaptic functions. These have been shown in various rodent models but direct measurements of excitatory–inhibitory changes have yet to be performed in human neurons. Therefore, our project aims to construct a human-induced pluripotent stem cell co-culture model representing important brain circuitry which captures synaptic dysfunction. Familial Alzheimer’s disease patient induced pluripotent stem cells carrying mutant *APP* V717I and their isogenic controls were differentiated into cortical glutamatergic neurons and astrocytes using dual-SMAD inhibition followed by *in vitro* corticogenesis. Building upon this, we differentiated inhibitory interneurons expressing parvalbumin and somatostatin via ventral patterning with sonic hedgehog activation. Then, we co-cultured these cells with differentiated cortical neurons and astrocytes. The properties of the co-culture model were validated using immunohistochemistry, confocal microscopy combined with electrophysiological whole-cell recordings. Confocal microscopy validated the presence of excitatory cortical neurons, astrocytes, and two inhibitory interneuron types, parvalbumin and somatostatin expressing interneurons within the co-culture. Whole-cell recordings revealed intrinsic membrane properties from individual excitatory and inhibitory neurons in this co-culture from day 70 onwards. Spontaneous synaptic activity recorded from the *APP* V717I-induced pluripotent stem cell model showed synaptic hyperexcitability correlated with altered morphological changes, which was expected in contrast to the isogenic control co-culture. Our novel co-culture model, including astrocytes, excitatory and inhibitory neurons, represents a strong model of brain circuitry in Alzheimer’s disease. These models will enable investigation of Alzheimer’s disease causative mutations on neuronal connectivity in human neurons allowing for confirmation of network dysfunction in Alzheimer’s disease in human neurons. It also has the potential of becoming a valuable preclinical tool to screen novel targeted therapies. This is a methods paper validating a human-induced pluripotent stem cell-derived excitatory–inhibitory neuron–astrocyte co culture with electrophysiological readouts and immunostaining, focusing on Alzheimer’s disease relevant network physiology.

## Teaser

Li *et al*. describe the development and validation of a novel *in vitro* human dementia model, a patient derived induced pluripotent stem cell co-culture containing astrocytes, excitatory and inhibitory neurons. The system reproduces an 87:13 excitatory–inhibitory cell ratio, and Alzheimer’s disease co-cultures showed distinct phenotypic and functional changes versus controls.

## Graphical Abstract

**Graphical Abstract:**
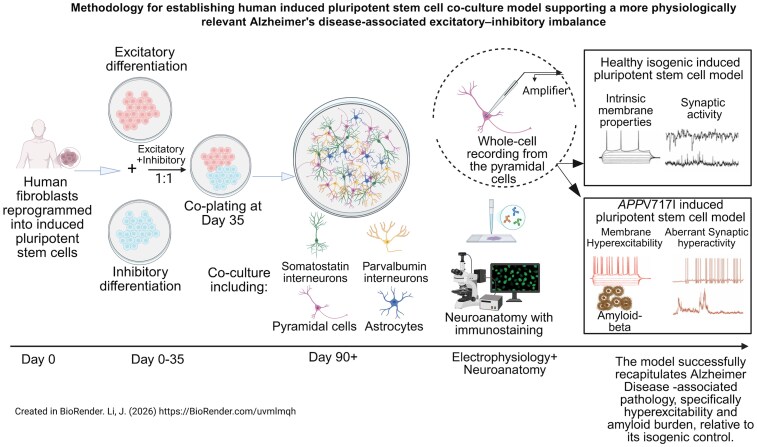

## Introduction

Alzheimer’s disease is the most prevalent neurodegenerative disease.^1-3^ The symptoms include gradual cognitive decline and progressive loss of connections between neurons and astrocytes, which we have shown to be associated with disrupted synaptic imbalance and excess inhibitory neurotransmitter GABA in rodent models at an early stage of Alzheimer’s disease.^4-6^ Current disease-modifying treatments for Alzheimer’s disease show modest effects at early stages of the disease, and this therefore represents a huge unmet clinical need. This challenge could be partially addressed by developing improved preclinical rodent models; however, it has been challenging to capture the main pathologies of Alzheimer’s disease coupled with neurodegeneration, particularly the selective vulnerability of human neurons,^7-9^ in a single rodent model, without relying on the exogenous expression of multiple mutant genes.^2,7,8^ This suggests that human neurons may be selectively vulnerable to disease insults and highlights the importance of developing human neuronal models of Alzheimer’s disease.^10-12^

Thus, there is a need to understand whether these phenotypes are present in human neurons, and this can be achieved through the use of patient-derived induced pluripotent stem cells (iPSCs), which enable a near limitless supply of human neurons to be generated *in vitro*.^13^ Furthermore, ethical concerns remain significant in the use of laboratory animals.^14,15^ In 2023 alone, ∼600 000 laboratory mice were used in dementia research.^16,17^

**Figure 1:**
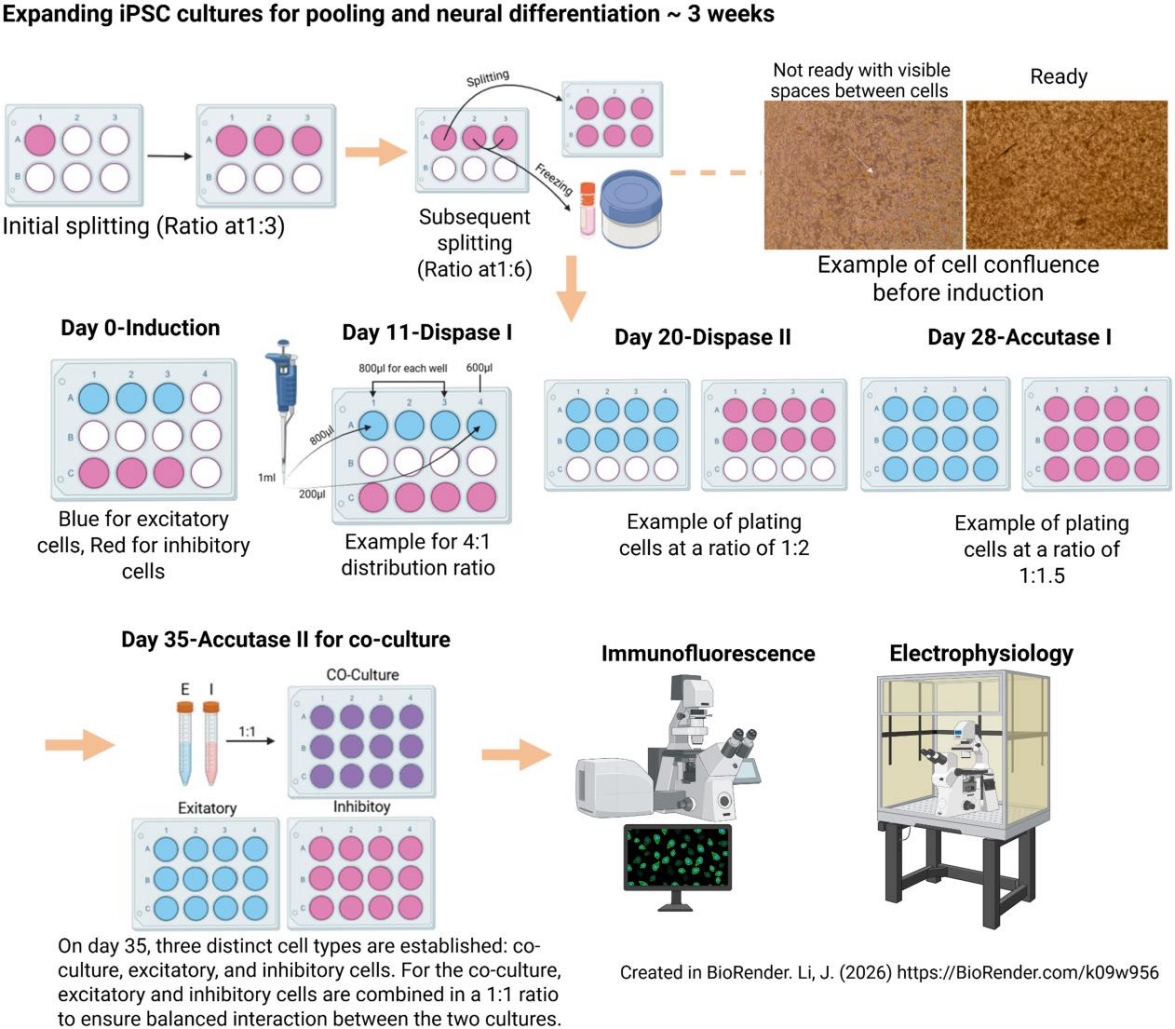
**Schematic representation of the iPSC co-culture method.** Stepwise expansion and differentiation protocol using splitting and enzymatic passaging ratios and optimized differentiation media to establish balanced neuronal co-cultures by Day 35. The resulting neuronal networks are analysed through immunofluorescence and electrophysiology. It suggests that this co-culture method provides a reliable platform to study neuronal network function and dysfunction in vitro. Created in BioRender. Li, J. (2026) https://BioRender.com/k09w956.

Therefore, this study aims to develop a fit for purpose system, an *in vitro* iPSC-based dementia model, by building a cortical co-culture from patient-derived human stem cells including excitatory neurons, multiple subclasses of inhibitory neurons, and cells that support neuronal function, namely astrocytes. While previous studies have established co-culture models containing both excitatory and inhibitory neuronal populations, our novel co-culture system is the first to specifically incorporate familial Alzheimer’s disease patient-derived excitatory and inhibitory neurons.^18-20^ This approach (Fig. 1) uniquely enables us to closely mimic human Alzheimer’s disease pathology, addressing critical unanswered questions in Alzheimer’s disease pathogenesis, improving scientific outcomes, and facilitating the gradual ‘Replacement and Reduction’ of laboratory research animals used for moderate-to-severe experimental procedures in dementia research.

## Materials and methods

This study utilized human iPSCs with an amyloid precursor protein (APP) V717I mutation, alongside its corresponding isogenic control. Fibroblasts from a female donor were reprogrammed in the laboratory of Prof. Selina Wray, as described previously.^1,2,9^

### Institutional review board statement

iPSC lines used in this study were generated in-house at UCL Institute of Neurology from dermal fibroblasts donated under written informed consent. Tissue procurement and iPSC reprogramming were approved by the Joint Research Ethics Committee of University College London Hospitals NHS Foundation Trust (National Hospital for Neurology and Neurosurgery) and the UCL Institute of Neurology joint research ethics committee (REC 09/0272), and that all donors provided written informed for research use. The *APP* V717I iPSC line corresponds to ‘APP patient 2’ as previously reported.^1^ All lines were deidentified prior to investigator access. No new human samples were collected for the present work. All procedures complied with institutional guidelines and the Declaration of Helsinki.

### Informed consent statement

Informed consent was obtained from all subjects involved in the study.

#### iPSC culture

iPSCs were thawed from cryopreservation and initially plated in Essential 8 (E8) medium (Life Technologies, UK) supplemented with Y-27632 ROCK inhibitor (Tocris Bioscience, UK), then cultured in Geltrex-coated 6-well plates (Life Technologies, UK). Medium changes were performed daily until the colonies reached ∼70% confluence. Upon reaching this confluence, cells were detached using a 0.5 mM EDTA solution (Life Technologies, UK) and split at ratios of 1:3 initially and 1:6 for subsequent passages. For long-term storage, cells were resuspended in neural freezing medium [10% Dimethyl sulfoxide (DMSO) in E8 medium at 1:10 ratio] and sequentially stored at −80°C in a Mr. Frosty freezing container overnight before being transferred to liquid nitrogen for cryopreservation.

#### Induction of iPSC-derived neurons

At 100% confluence, iPSCs were transferred to Geltrex-coated 12-well plates and grown again to full confluence. Neural induction was then initiated by replacing the E8 medium with induction media specific to either excitatory or inhibitory neuron differentiation as detailed below:

##### Excitatory neuronal induction

Excitatory neural induction medium consisted of N2B27 neuronal maintenance media (see Table 1 for detailed reagents) supplemented with 10 µM SB431542 (Tocris Bioscience, UK) and 1 µM dorsomorphin (Bio-Techne, UK). This medium was refreshed daily for 10 days to promote differentiation. On Day 11, neuroepithelial sheets were isolated using Dispase solution (Life Technologies, UK) and transferred onto laminin-coated plates (Sigma, UK). Cells were subsequently split at 3:4 ratio and maintained in neural maintenance medium N2B27, with media changes every other day. Between Day 12 and 18, neuroepithelial rosettes formed, expressing neural tube-specific proteins.

**Table 1:** N2B27 neuronal maintenance media

Reagent	Purpose	For 500 mL volume
Neurobasal media	For long-term growth and viability of postnatal and adult hippocampal and cortical neurons.	250 mL
DMEM/F12 Glutamax	Foundational basal medium containing nutrients for neuronal differentiation and maintenance	250 mL
N2B27 supplements		
N2 supplement	Growth of neuronal cell types	2.5 mL
Non-essential amino acid	Raw material for cellular proteins	2.5 mL
2-mercaptoethanol	HOCH_2_CH_2_SH. antioxidant	0.5 mL
Pen strep	Antibiotic	2.5 mL
Insulin	For uptake and utilization of glucose, amino acids and lipids.	125 µL
B27	Used to support the low- or high-density growth and short- or long-term viability of embryonic, post-natal and adult hippocampal and other CNS neurons.	5 mL
L-glutamine	For glutaminolysis i.e. cellular energy	2.5 mL

##### Inhibitory neuronal induction

Inhibitory neural induction medium was composed of N2B27 neuronal maintenance media supplemented with 10 µM SB431542 (Tocris Bioscience, UK), 100 nM ALK2/3 inhibitor LDN-193189 (Tocris Bioscience, UK), and 2 µM tankyrase inhibitor XAV939 (Cayman Chemical Company, UK). On Day 11, neuroepithelial sheets were isolated using Dispase solution and transferred onto laminin-coated plates. Cells were split at a 3:4 ratio and cultured in neural maintenance medium N2B27, supplemented with 5 nM recombinant mouse Sonic Hedgehog/Shh (C25II) N-Terminus (Bio-Techne, UK) and 1 µM Stemolecule™ Purmorphamine (AMSBIO, UK). Media were refreshed every other day to support differentiation. Between Day 12 and 18, neuroepithelial rosettes formed, expressing neural tube-specific proteins. Because PV and SST emerge late in interneuron maturation, we interpret them as evidence of inhibitory identity at later time points, rather than as confirmation of early lineage commitment.

##### General culture method

From Day 20 to 35, excitatory and inhibitory neuronal cultures were maintained under identical conditions while continuing to be cultured separately. On Day 20, Dispase solution was used again to further purify and expand the cultures at a 1:1 or 1:2 ratio, depending on culture density. The cells were fed with neural maintenance medium N2B27 every other day. By Day 28, cells were detached using Accutase medium (Life Technologies, UK), pooled, and re-plated at a ratio of 1:1.5 to promote growth and maturation in neural growth media, which is used to induce proliferation and differentiation of neural precursor cells. The medium is prepared by neural maintenance medium N2B27 supplemented with 1 ngml^−1^ BDNF (R&D Systems, UK), 1 ngml^−1^ GDNF (Bio-Techne, UK) and 6 μM dbcAMP (Sigma, UK), which was replaced every 2 days. The necessary number of co-cultures and single-cell cultures for excitatory and inhibitory neurons should be planned and established in advance. On Day 35, excitatory and inhibitory neuronal cultures were combined to establish co-cultures. Cells were detached and pooled into falcon tubes, with cell density determined using a cell counter (Countess 3, Life Science, UK). Single-cell cultures were replated at a minimum density of 50 000 cells per cm^2^, while co-cultures were replated at 30 000 cells per cm^2^, combining excitatory and inhibitory cells at a 1:1 ratio. The cultures were then fed every 3–4 days with neural growth media to support continued development, and every 2 weeks, the media was supplemented with laminin at a 1:200 dilution to help promote cell attachment to coverslips. All long-term cultures were inspected for contamination routinely and tested for mycoplasma monthly.

### DNA extraction

DNA was extracted from snap-frozen iPSC pellets using Qiagen DNeasy kit (Qiagen, NV) according to the manufacturer’s instructions including all optional steps. DNA was quantified and quality-assessed using nanodrop prior to use in downstream applications.

### Mycoplasma testing

Media supernatant from iPSC lines underwent mycoplasma testing using Eurofins’ MycoSEQ^TM^ assay (Eurofins Scientific).

### Karyotyping and sanger sequencing

Karyotyping analysis was conducted using Stem cell Technologies Human Pluripotent Stem Cell (PSC) Genetic Analysis qPCR kit (STEMCELL Technologies, UK) using the AriaMx Real-Time PCR system. Sanger sequencing was carried out by UCL Genomics on isolated genomic DNA.

### Immunofluorescence

Cells were fixed in 4% paraformaldehyde for 15 min and stored in phosphate buffered saline (PBS) at 4 °C until immunostaining was performed. Prior to the specific procedures, cells were pretreated by incubating in 0.3% Triton X-100 in PBS for 30 min. Cells were rewashed in 0.1% PBS-Tween (PBS-T) before being treated in 5% bovine serum albumin (Sigma-Aldrich, USA) in PBS-T for 1 h at room temperature. The following incubation with primary antibodies are reported in Table 2 to target specific proteins. After a 24-h incubation in a primary antibody at 4°C and three washes in PBS-T, cells were then incubated in appropriate secondary antibodies for 1 h at room temperature (immunofluorescence fluorophores are listed in Table 2). After three 5-min washes in PBS-T, cells were incubated with DAPI for 15 min. To prepare the slices for confocal microscopy, all the cells were washed once more before being placed on glass slides with antifade mounting solution Vectashield (Vector Labs, UK). Images were captured at room temperature on a ZEISS LSM 710 microscope at ×40, and ×63 magnification using Zen Black 2009 software. No *post hoc* manipulation was performed.

**Table 2:** Antibodies used for immunofluorescence and target cell marker identification

Antibody name	Antibody target(s)	Species	Dilution	Supplier	Cat. No.
Anti-Pax 6	Neuroectodermal cells and early neural progenitor cells	Rabbit	1:500	Bio-Techne	NBP1-89100
MAP2	Neuron dendrites and neuronal differentiation/maturation	Chicken	1:2500	Abcam	AB92434
FOXG1	Early neural progenitor cells destined to form the cerebral cortex	Rabbit	1:300	Abcam	AB12859
NKX2-1	Transcription factor necessary for the specification of interneurons	Mouse	1:300	Invitrogen	MA5-13961
TBR2	Intermediate progenitor cells or basal progenitors in the developing cortex.	Rabbit	1:200	Abcam	AB23345
TBR1	One of the earliest markers of cortical neuron identity	Rabbit	1:300	Abcam	AB31940
VGLUT 1	Check the differentiation process with glutamatergic neurons	Rabbit	1:300	System	135303
Anti-Somatostatin (SST)	Identify specific subtypes of inhibitory GABAergic interneurons	Rat	1:500	Merk	MAB354
Anti-Parvalbumin (PV)	Interneurons that maintain a balance of excitatory and inhibitory signals in the brain	Mouse	1:1000	Thermo Fisher Scientific	MA5-45927
CAMKII	Indicate the successful differentiation of the iPSCs into mature neurons.	Goat	1:100	Invitrogen	PA5-19128
GFAP monoclonal	Reactive astrocytes	Mouse	1:1000	Agilent (Dako)	13-0300
Wisteria floribunda agglutinin (WFA)	Structural support	Biotinylated	1:400	Vector Laboratories	B1355-2
SOX9	Astrocytes	Rabbit	1:1000	Abcam	AB185966
NANOG	Nuclear pluripotency marker	Rabbit	1:500	Cell Signalling Technology	4903s
SSEA4	Surface pluripotency marker	Mouse	1:250	BioLegend	330401
Secondary antibody					
Alexa 488		Rabbit	1:1000	Abcam	AB150125
Alexa 555, streptavidin		Biotinylated	1:400	Thermo Fisher Scientific	S21381
Alexa 568		Rat	1:1000	Abcam	A11077
Alexa 568		Rabbit	1:1000	Abcam	AB175707
Alexa 647		Goat	1:1000	Abcam	AB150115
Alexa 647		Mouse	1:1000	Abcam	AB150115
DAPI		Multiple	1:2000	Sigma-Aldrich	D9542

### Media transition protocol for electrophysiology

All iPSC-derived cultures were transitioned to BrainPhys^TM^ neuronal medium supplemented with SM1 (Stemcell Technologies, UK) 2 weeks prior to electrophysiology experiments, to support basic synaptic function and activity.^3,4^ During the first week, cultures underwent half-media changes with 500 µl supplemented BrainPhys^TM^ media and 500 µl neural growth media biweekly to allow cells to acclimatize, before being fed with supplemented BrainPhys^TM^ alone thereafter.

### Electrophysiology

Whole-cell recordings were performed under near-infrared differential interference contrast (DIC) microscopy using an upright microscope (Leica, Germany), which facilitated the visualization of target cells on a monitor (Panasonic, UK).

For this procedure, patch electrodes made from filamented borosilicate glass capillaries (Harvard Apparatus, UK) with resistances of 8–11 MΩ were filled with ACSF solution containing (in mM) 121 NaCl, 2.5 KCl, 1.3 NaH2PO4, 2 CaCl2, 1 MgCl2, 20 glucose, 26 NaHCO3 and Biocytin, equilibrated with 95% O2 and 5% CO2 (pH, 7.3, osmolarity, 300–310 mOsm). Glass micropipettes were attached onto a portion of the cell membrane, which was then ruptured with suction to provide electrical access to the intracellular space and allow biocytin influx into the cell.

Recordings were carried out under the current clamp mode of operation using either NPI SEC 05LX amplifier (NPI electronics, Germany) or Multiclamp 700B amplifier (Molecular Devices), where data were low pass filtered at 2KHz and digitized at 5KHz using a CED 1401 interface (Cambridge Electronic Design, UK). Intrinsic electrophysiological properties were obtained from injecting 500 ms hyperpolarizing and depolarizing current pulses (−50 pA to +150 pA in 25 or 50 pA increments). Spontaneous postsynaptic potentials were recorded from passive membrane responses and mixed spontaneous excitatory postsynaptic potentials (sEPSPs) and spontaneous inhibitory postsynaptic potentials (sIPSPs) were collected in 60 s frame samples, repeated at 0.33 Hz. After recording electrophysiological recordings of neurons, *post hoc* labelling of the recorded neuron with streptavidin for subsequent anatomical protocols were performed.^4^ Images during the procedure were enhanced using a camera control unit (Hamamatsu, Japan).

Action potentials, resting membrane potentials and the current–voltage relationships were quantified using custom MATLAB scripts, where peaks with a prominence greater than 5× the baseline noise were considered as events. A 20 ms window around each action potential was excluded from mean resting membrane potential calculation. All detected events were visually verified. Cells with a holding current lower than −100 pA were excluded from analysis.

### Morphometric analysis

Following biocytin labelling, immunofluorescence and horseradish peroxidase (HRP) staining of pyramidal cells from both iPSC co-cultures, anatomical reconstruction of all cells was performed manually using a drawing tube attached to a light microscope (Leica DMR, Germany) under a ×40 objective lens. Sholl analysis was performed from hiPSC-derived pyramidal cells (*APP* V717I and isogenic) using the ImageJ (Version 1.54, RBS, MD, USA) Sholl analysis Plug-in, and a radius step size of 10 μm.

### Statistical analyses

Statistical analyses accounted for both patient-derived cell pairs and isogenic biological replicates to ensure robustness and reproducibility. Each experiment utilized matched pairs comprising Alzheimer’s disease patient-derived cells and their corresponding isogenic controls. Biological replicates were independently cultured from identical iPSC lines to accommodate variability inherent in cell culture techniques.

Fluorescence intensity was quantified using FIJI/ImageJ (Version 1.54). Cultures with excessive beading/cell debris and/or had confluency <30%, indicative of very poor viability, were not used for analysis to prevent sampling bias. For each coverslip, 10 non-overlapping 40× fields were acquired using ZEISS LSM 710 microscope across genotypes within a given experiment. Background was subtracted and integrated fluorescence density for each marker was measured and normalized to the number of DAPI-positive nuclei per field. Fields were selected according to a predefined sampling pattern that was independent of signal intensity, and images were coded so that the analyst was blinded to genotype during quantification.

Statistical analyses were performed using GraphPad Prism (version 9.0 for Windows and version 10.4.1 for Mac) and Microsoft Excel. Preliminary analyses comparing isogenic and *APP* V717I datasets indicated that a sample size of *n* ≥ 3 (from independent inductions, although two different lines were used) was sufficient to achieve over 80% statistical power at a 5% significance level, employing a two-sided *t*-test for immunofluorescence experiments. Immunofluorescence experiments involved quantitative analyses conducted across 10 distinct regions per iPSC sample, enhancing data reliability and accuracy.

In figures, error bars represent variability as indicated, and ‘*n*’ denotes the number of observations, typically reflecting the number of animals or human patients, unless otherwise specified. Statistical significance was determined using a 95% confidence interval, with significance set at *P* ≤ 0.05. Morphometric analyses of parameters including the number of branch points, number of branches, dendritic length and Sholl profiles were assessed using two-way ANOVAs with Sidak’s multiple comparisons test.

Outliers were identified using 2 SD criteria applied at the line-mean level and excluded prior to statistical testing. Normality of per-line data was assessed using the Shapiro–Wilk test in combination with Q–Q plots. All non-normally distributed data were presented as median and interquartile range, whereas normally distributed data were presented as mean and standard error of the mean. All statistical tests were carried out on the most accurate measure of central tendency from each line (i.e. mean or median), and unpaired t-tests were used for comparing normally distributed data whereas Mann–Whitney *U* test was used for non-normally distributed data. Two-way ANOVA with Sidak’s multiple comparisons test was used to analyse current–frequency relationships. All statistical analysis for electrophysiology results is preliminary due to limited number of technical replicates.

## Results

### Characterization of iPSCs

**Figure 2:**
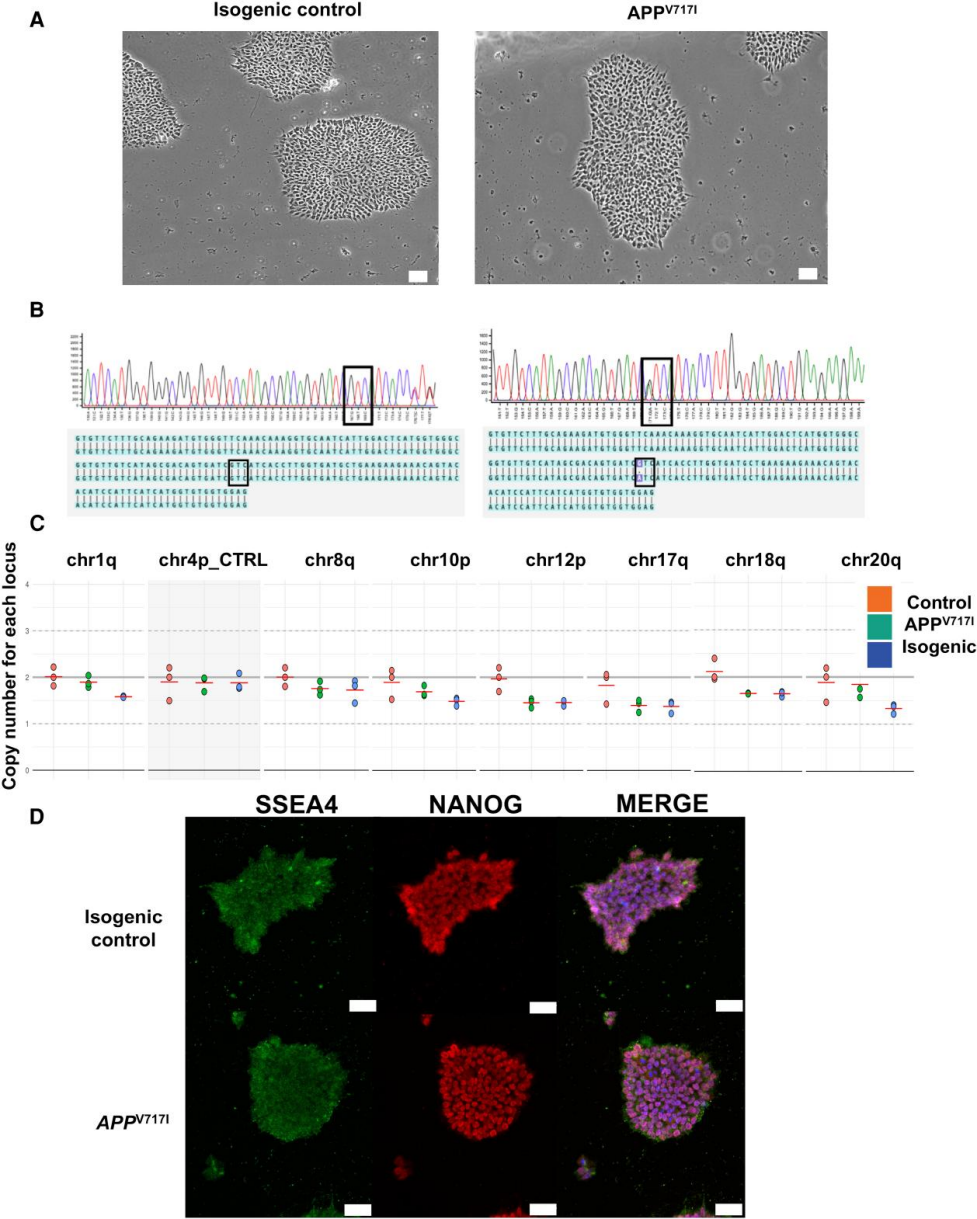
**Quality control check of iPSC lines derived from an *APP* V717I patient and isogenic control.** (**A**) Digital brightfield imaging of iPSC stem cell colonies from *APP* V717I and isogenic control lines. Scale bar = 50 µM, ×20 magnification. (**B**) Sanger sequencing results from extracted DNA from both lines (performed by SourceBio). (**C**) Karyotype results, displayed using Stem Cell Technologies hPSC Genetic Analysis tool, show no significant difference in copy numbers in the APP V717I line (median *P* = 0.18–0.99) and its isogenic control (median *P* = 0.14–0.96) at each locus compared to a standardized control. This was carried out on the two lines, where each point represents a technical replicate. (**D**) Immunofluorescence staining of pluripotency markers SSEA4 (Alexa Fluor 488) and NANOG (Alexa Fluor 594) in both lines. Scale bar = 50 µM, ×40 magnification.

iPSC lines underwent quality control checks before each neuronal induction to validate their identity, genomic integrity and pluripotency. Digital brightfield imaging showed iPSCs formed compact, rounded colonies following thawing and passaging, indicative of healthy and unstressed cells (Fig. 2A). Sanger sequencing carried out on DNA isolated from the cells confirmed the expected genotype for both lines, where *APP* V717I cells possess the characteristic missense mutation (G > A), which is absent in the isogenic line (Fig. 2B). Genomic integrity was assessed using the human pluripotent stem cell genetic analysis qPCR kit (StemCell Technologies) and did not show detectable copy number variations in the most common locations of genomic instability (Fig. 2C). Lastly, pluripotency was determined using immunocytochemistry, where colonies stained positively for common pluripotency markers SSEA4 and NANOG (Fig. 2D). Finally, both lines were negative when sent for mycoplasma testing.

### Timeline and changes in morphological characteristics of iPSCs and neural rosettes

Shi *et al*. have previously described the differentiation timeline of human cortical neurons derived from iPSCs. Day 1–10, early neural differentiation. Day 12–19, high density colonies form neural rosettes. Day 12–17, early born deep layer 6 projection neurons and neural precursors form. Day 29–35, formation of differentiating neurons expressing TuJ1 and post-mitotic projection neurons expressing TBR1. From day 30–50, synaptic connections begin to form, and neurons mature. The majority of cells in this timepoint are early born deep layer 6 neurons. Astrocytes begin to emerge from day 90+, as confirmed by our data. Day 75 + late born upper layer neurons expressing SATB2/CUX1 begin forming and synaptic networks reach functional maturity. Day 90 + upper layer neurons fully mature, representing the conclusion of the differentiation and maturation process.^5,6^

**Figure 3:**
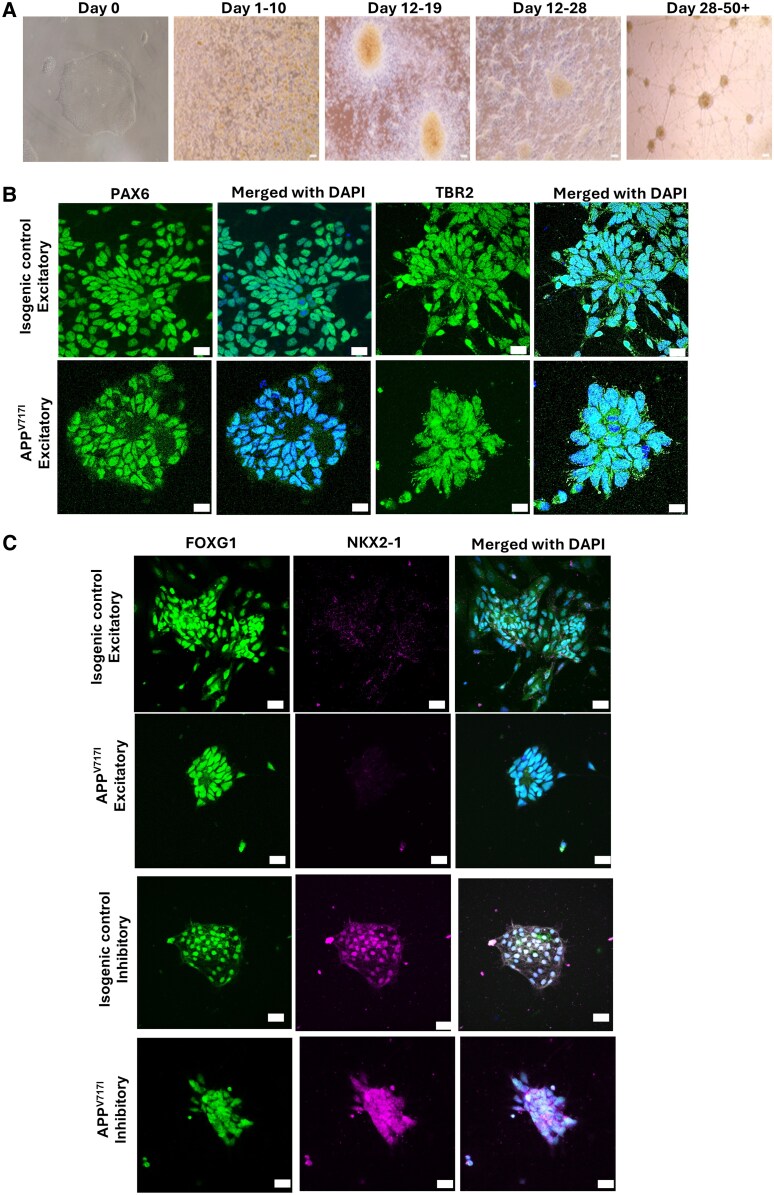
**Morphological changes in iPSCs during differentiation and confocal microscopy immunofluorescence staining of iPSC-derived cortical stem cells/progenitors.** (**A**) Cells undergo morphological changes at distinct timepoints. (**B**) Day 21 excitatory and inhibitory neuron progenitor cultures with isogenic and *APP* genotypes. FOXG1 and PAX6 denote primary cortical progenitor cells. TBR2 represents secondary cortical progenitor cells. Scale bar = 50μm, ×40 magnification. FOXG1, TBR2, PAX6 (Alexa fluor 488 green). This suggests that the *APP* mutation does not significantly alter early cortical progenitor identity at day 21 but may affect subsequent neuronal differentiation and maturation stages. (**C**) Day 28 excitatory and inhibitory neuron progenitor cultures with isogenic and *APP* genotypes. NKX2-1 (Alexa fluor 647 pink) denotes interneuron progenitors. Scale bar = 50μm, ×40 magnification.

Our observations in Fig. 3 align with published corticogenesis timelines: iPSC colonies progress to neuroepithelial rosettes (Days 12–19), followed by differentiating neurons and network maturation (Days 30–50+). Immunostaining at Day 21 showed FOXG1 and PAX6 in primary cortical progenitors and TBR2 in intermediate/basal progenitors, consistent with dorsal cortical lineage progression; these markers do not specify inhibitory (ventral) fate. Inhibitory interneuron commitment in our workflow was induced by SHH/Purmorphamine ventral patterning, which showed early ventral marker *NKX2-1*. More extensive characterization of ventral progenitors was not included (*LXH6, DLX1.2*) and instead, later-stage evidence of inhibitory differentiation is provided by the appearance of SST- and PV-positive interneurons at subsequent time points.

### Excitatory and inhibitory cortical neurons form in cortical neuron co-cultures *in vitro*

The major enhancement in the *in vitro* corticogenesis protocol is the ability to generate distinct populations of excitatory and inhibitory cortical neurons separately and then combine them in co-culture. To facilitate the study of excitatory/inhibitory (E/I) interactions, excitatory and inhibitory neurons were separately differentiated, and then combined at a 1:1 ratio after 35 days *in vitro* (DIV), which results in an 87:13 E/I ratio, and 1:0.89 neuron/astrocyte ratio by DIV90 in the isogenic control line (Supp_Fig_1). As the human cortex consists of ∼80% excitatory neurons and 20% GABAergic inhibitory interneurons, including both populations was essential to create balanced synaptic networks in the co-culture system.

**Figure 4:**
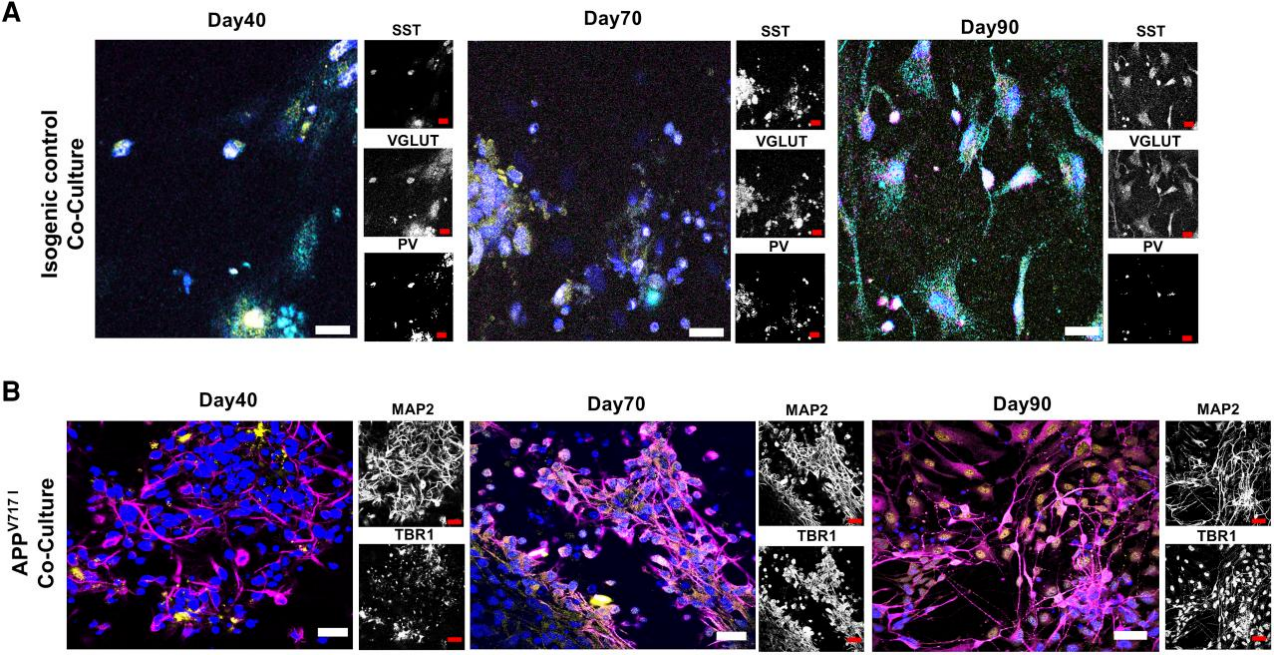
**Excitatory and inhibitory cortical neurons form in cortical neuron co-cultures.** Multiplex immunocytochemistry followed by Z-stack confocal microscopy images of cortical neuron co-cultures at distinct timepoints. Timepoints 40, 70 and 90 represent two combinations of co-staining: (**A**) VGLUT1 (glutamatergic neurons and their synapses), SST (somatostatin interneurons) and PV (parvalbumin interneurons). (**B**) MAP2 (neuronal dendrites) and TBR1 (cortical neurons). Scale bar = 50μm, ×40 magnification. This suggests that the progressive expression and co-localization of neuronal markers reflect successful maturation and integration of excitatory and inhibitory neuronal populations, modelling functional cortical network development over time.

Throughout major timepoints (day 40, 70, 90), TBR1 deep layer neurons stain positive, with a proportion of dendrites colocalizing with MAP2. VGLUT1 (vesicular glutamate transporter 1)-positive cortical neurons, an abundant type of cortical excitatory neuron, are contrasted with two subclasses of inhibitory interneurons: somatostatin (SST) and parvalbumin expressing interneurons. In particular, fast-spiking parvalbumin interneurons modulate the timing of action potentials and target the perisomatic region of pyramidal cells. Somatostatin-positive interneurons gate excitatory signals and target the dendrites of pyramidal cells. These interneurons maintain the cortical excitatory/inhibitory balance. In the Alzheimer’s disease state, amyloid plaques and oxidative stress contribute to the selective vulnerability of both subclasses of interneurons. The hypoactivity of PV cells leads to network hyperexcitability and SST cell impairment disrupts dendritic signal integration. Figure 4 illustrates these cell types at three timepoints.

### Astrocytes and reactive astrocytes form in homogenous excitatory cortical neuron cultures *in vitro*

Astrocytes, star shaped glial cells, perform a myriad of functions in the cortex. In the cortex, astrocytes form part of the tripartite synapse where they uptake synaptic glutamate and release gliotransmitters to modulate synaptic plasticity. In Alzheimer’s disease, astrocytes are also critical in the neuroinflammatory response. When astrocytes are activated, they undergo morphological, biochemical, and functional changes: form a glial scar and release proinflammatory cytokines. These reactive astrocytes are of key interest in Alzheimer’s disease models.^7^ As such, it was important to recapitulate both reactive and non-reactive astrocytes in our cortical neuron cultures.

**Figure 5:**
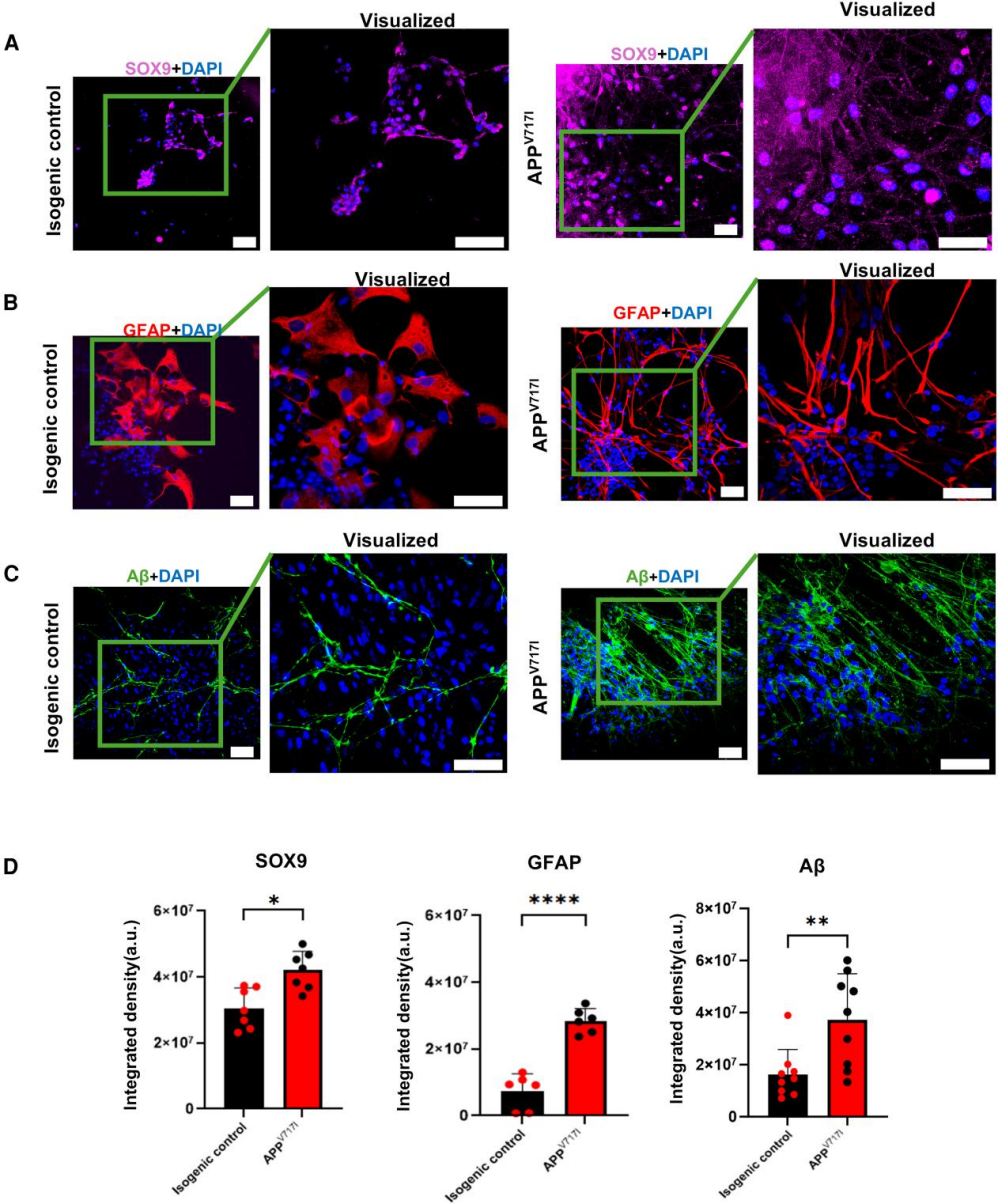
**Day-90 astrocytes and Aβ in human iPSC-derived cortical cultures.** (**A**) SOX9 (magenta) with DAPI (blue) in isogenic control (left) and *APP* V717I (right) cultures. (**B**) GFAP (red) with DAPI (blue) in the same conditions as **A**. (**C**) Aβ (green) with DAPI (blue) in isogenic (left) and *APP* V717I (right) cultures. Representative confocal Z-stack maximum projections. Scale bar = 50 µm; 40× objective. (**D**) Quantification of signal: integrated fluorescence density for SOX9 (astrocyte identity), GFAP (astrocyte reactivity), and Aβ, each normalized to the number of DAPI-positive nuclei per field (Student *t* test, *P ≤ 0.05 ** *P* ≤ 0.01, ***P ≤ 0.001, ****P ≤ 0.0001). This indicates that *APP* V717I mutations are associated with increased astrocyte reactivity and Aβ burden in *APP* V717I cultures compared with isogenic controls. Data were collected from 3 groups originating from 2 patient pairs, with non-overlapping fields captured from each coverslip and 3 coverslips per genotype per group, *n* = 7. Image acquisition and quantification were performed with the experimenter blinded to genotype.

We observed astrocyte formation around day 90, the timepoint at which neuronal networks were functionally mature, consistent with Shi *et al*.,^8^ who reported the gliogenic switch occurring after corticogenesis is complete. In both the *APP* V717I and isogenic genotypes, SOX9-positive astrocytes, GFAP-positive reactive astrocytes and amyloid beta (Aβ) were detected (Fig. 5A–C). SOX9 was used as a general astrocyte identity marker, whereas GFAP together with Alzheimer’s disease–relevant readout of Aβ burden was used to assess astrocyte reactivity. Quantification of DAPI-normalized integrated fluorescence density showed that, relative to isogenic excitatory cultures, *APP* V717I cultures had ∼33% higher SOX9, 200% higher GFAP and 137% higher Aβ signal per nucleus (Fig. 5D). Morphologically, GFAP^+^ astrocytes in *APP* V717I cultures displayed thicker, more highly branched processes than in isogenic controls, consistent with enhanced reactive astrogliosis in the *APP* V717I condition.

Together, these data show that our culture system not only supports astrocyte maturation and reactivity but also exhibits measurable Aβ deposition, anchoring the model to a core pathological hallmark of Alzheimer’s disease.

### Intrinsic membrane properties recorded at days 30, 70 and 95

**Figure 6:**
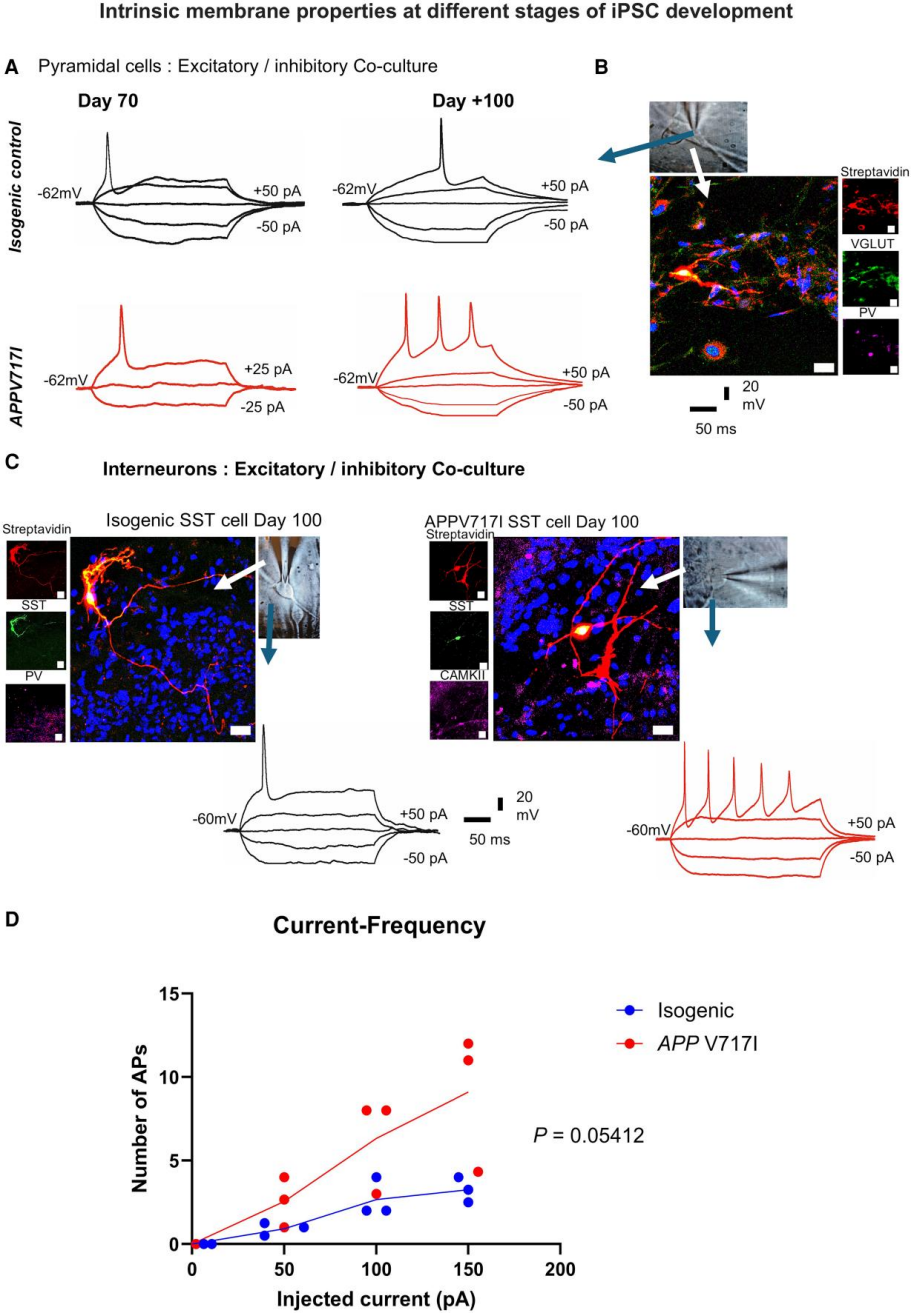
**Intrinsic membrane properties become more complex with maturity and more hyperexcitable in the *APP* V717I line compared to isogenic line.** (**A, B**) Intrinsic membrane properties obtained from whole-cell recordings from cells in ISO co-culture. Example biophysical properties of PV-expressing interneuron and CAMKIIα glutamatergic cell recorded under differential interference contrast (DIC) microscopy (black/white inserts under ×40). The recorded cells were stained *post hoc* and recovered using a streptavidin protocol. (**C**) Example biophysical properties of SST-expressing interneuron recorded under differential Interference Contrast microscopy (black/white inserts under ×40). The recorded cells were recovered using a streptavidin protocol (streptavidin recovers neuronal morphology). The glutamatergic cell (CAMKIIα) and all interneurons (SST) elicited more action potentials in the *APP* V717I models compared to the Isogenic controls with the same amount of current injected. (**D**) Relationship between injected current and number of action potentials fired in *APP* V717I and isogenic controls. Firing increased with current injections (repeated-measures ANOVA, *P* = 0.0072) and trended towards being higher in *APP* V717I neurons overall (*P* = 0.0541). No significant interaction was observed between current and genotype (*P* = 0.1058) indicating that the genotype difference was consistent across all current injections. Bold line signifies mean AP frequency at each current step. *APP* V717I and Isogenic were pooled from three independent differentiations where each point represents the mean from each differentiation (*n* = 5 or more cells per mean), *P < 0.05.

Electrophysiological whole-cell patch-clamp intracellular recordings were performed to distinguish the biophysical properties of the glutamatergic and GABAergic neurons as their active action potential firing and other membrane properties is expected to be specific to the class of neuron recorded shown in Fig. 6.^9^ The recorded cells were recovered anatomically to validate and study their morphology as described below and shown in Fig. 6B and C. The typical membrane potential of neurons recorded from the excitatory/inhibitory *APP* V717I co-culture was −58 mV compared with −62 mV for their isogenic control. The intrinsic membrane properties displayed improved with age, i.e. the action potential and after hyperpolarization properties became more complex from DIV 70 to DIV +100 (Fig. 6A). The electrical properties recorded from pyramidal cells and interneurons between DIV 30 and 70 were not displayed due to the immaturity of the cells. Interestingly, both pyramidal cells and interneurons from *APP* V717I cultures showed a trend towards increased firing compared with isogenic controls (*P* = 0.05412); however, no significant interaction was observed between current and genotype at the current sample size (*P* = 0.1058) (Fig. 6A and D).

Pyramidal cells recorded from both genotypes displayed more elaborate membrane properties beyond DIV 95 compared to DIV 75 homologues; however, it is noteworthy to mention that generally the properties of pyramidal cells were more distinguished in the mixed co-culture rather than the excitatory/astrocyte culture.

### Spontaneous activity of iPSC co-culture

**Figure 7:**
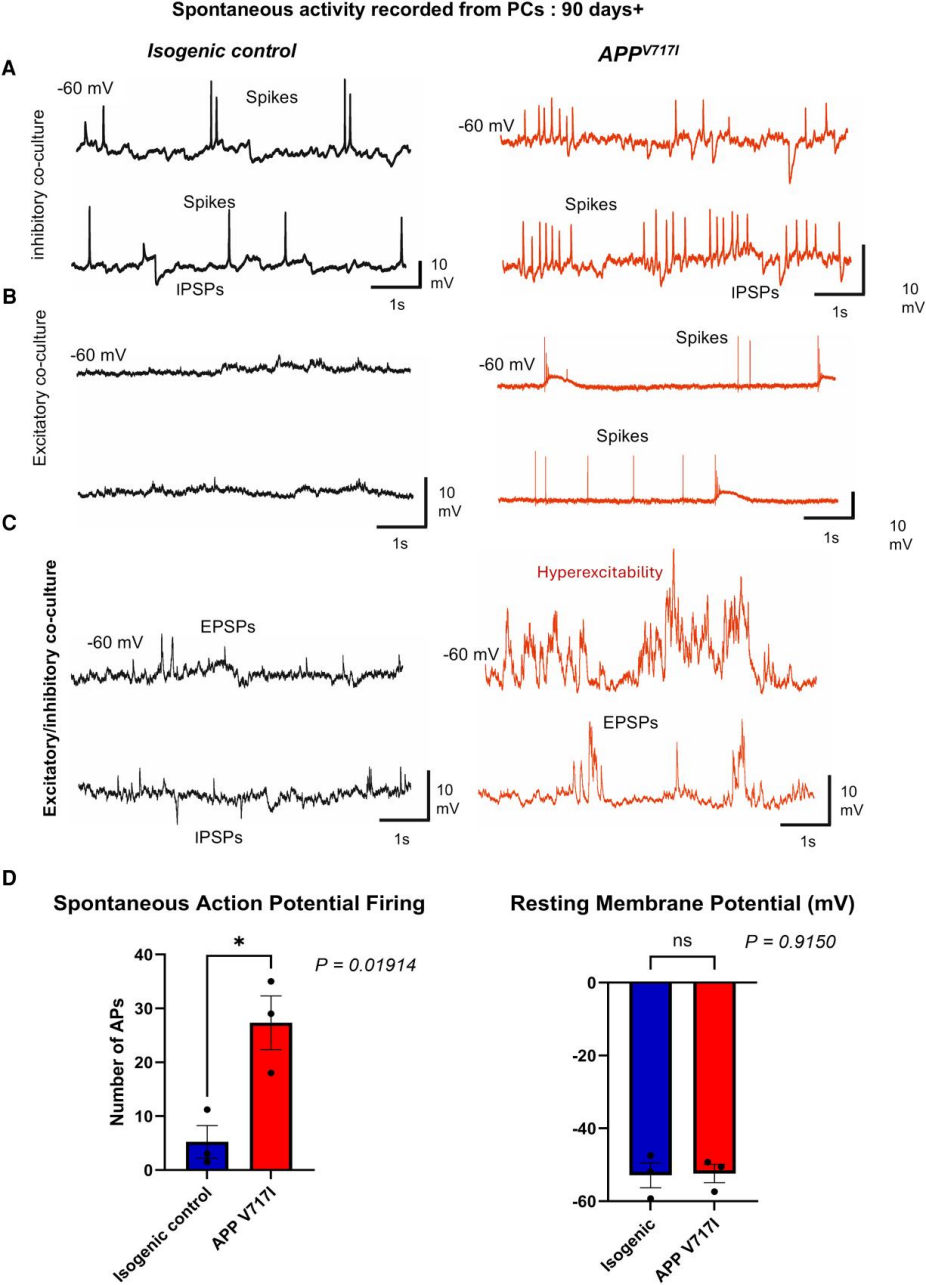
**Electrophysiological recordings indicate aberrant synaptic hyperexcitability in *APP* V717I excitatory/inhibitory co-culture.** (**A–C**) Example traces of whole-cell patch clamp recordings under current-clamp without current applied from DIV 90+ *APP* V717I and ISO excitatory-astrocyte, inhibitory-astrocyte and excitatory/inhibitory co-cultures. Inhibitory post synaptic potentials (IPSPs), excitatory post synaptic potentials (EPSPs) and spiking labelled. (**D**) (Left) Number of spontaneous action potentials within a 60 s window from DIV 90+ *APP* V717I iPSC-derived neurons and isogenic controls. (Right) Resting membrane potential from DIV 90+ *APP* V717I iPSC-derived neurons and isogenic controls. Bar graph presents the mean and standard error of the mean. Preliminary statistics for comparison of the mean spontaneous action potential firing and mean resting membrane potential were unpaired t-tests. * *P* < 0.05. *APP* V717I *n* = 6 (cells), Isogenic *n* = 8 (cells) pooled from three independent differentiations per line, where each point represents the mean from each differentiation.

Previous studies have shown synaptic imbalance in rodent models of Alzheimer’s disease, resulting in increased synaptic excitability and spontaneous firing.^4^ To evaluate whether this is recapitulated in our model, whole-cell patch clamp recordings were conducted in current-clamp without current applied. In line with previous findings, both individual *APP* excitatory and inhibitory neuron-astrocyte co-cultures in addition to excitatory/inhibitory co-cultures showed increased spontaneous firing compared with isogenic controls, indicative of hyperexcitability (*P* = 0.01914) (Fig. 7A–D).

### *Post hoc* recovery of recorded cells and morphometric analysis

**Figure 8:**
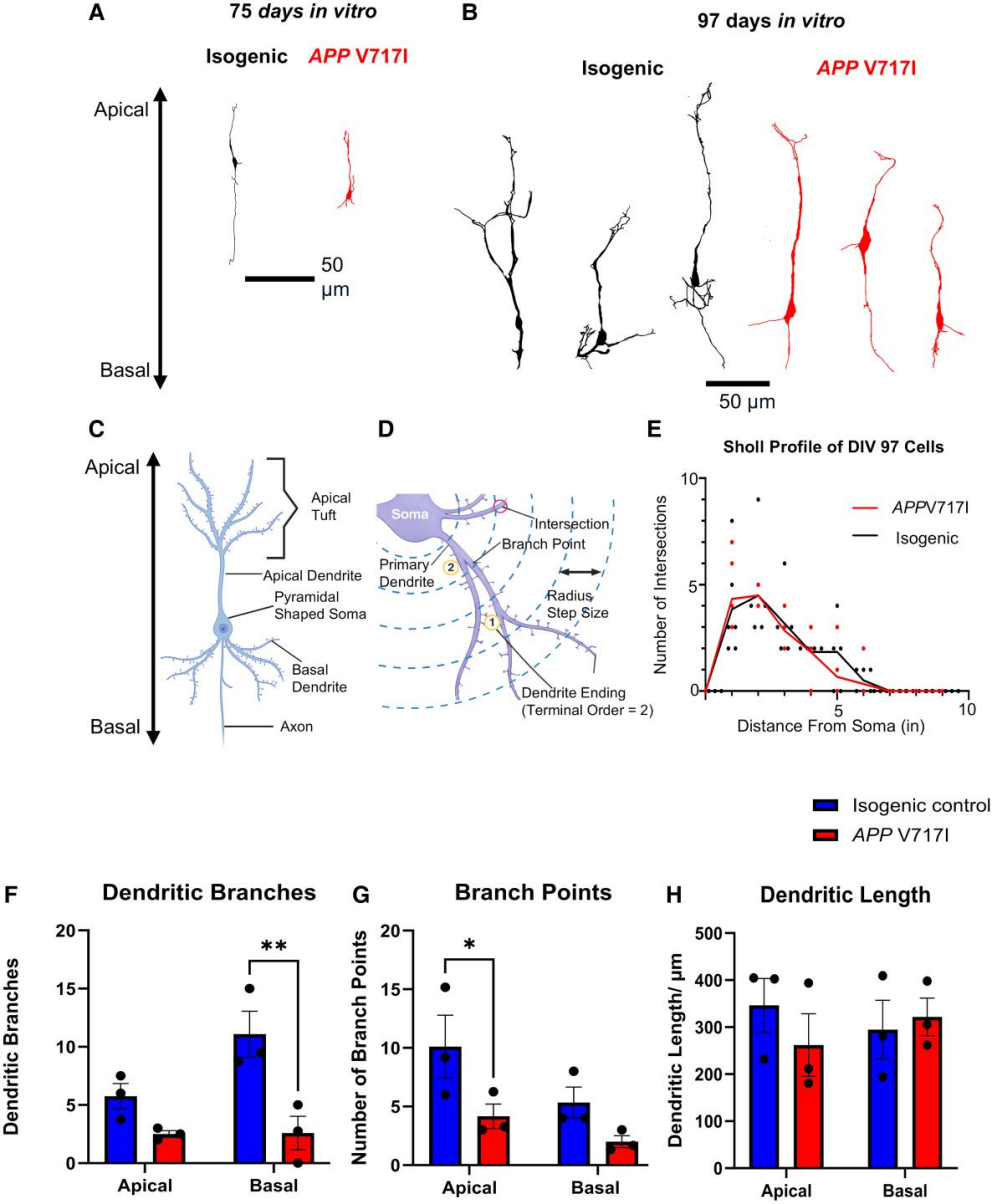
***APP* V717I iPSC-derived pyramidal cells exhibit reduced dendritic complexity compared to isogenic controls.** (**A** and **B**) Representative pyramidal cells at DIV 75 and DIV 97 demonstrate fewer dendritic branches and points in *APP* V717I cells, although typical pyramidal morphology is retained. (**C, D**) Diagrams illustrate pyramidal neuron structure and Sholl analysis method. (**E**) Sholl profile from pyramidal cells from DIV 97 cultures shows the mean number of intersections at a set distance from the soma and each point represents the number of intersections from a single cell at the corresponding distance (*APP* V717I *n* = 7, isogenic, *n* = 6), no statistics was performed. (**F, G**) Two-way ANOVA with Sidak’s multiple comparisons test indicate a significant reduction in the number of basal dendritic branches in *APP* V717I cells (*P* = 0.0083) but not apical dendritic branches (*P* = 0.4166) compared to control. There was also a significant reduction in the number of apical dendritic branch points between genotypes (*P* = 0.0310) but not basal branch points (*P* = 0.1811). (*APP* V717I pyramidal cells *n* = 9, isogenic pyramidal cells *n* = 14) (**H**) Two-way ANOVA with Sidak’s multiple comparisons test showed no differences in apical (*P* = 0.7985) or basal (*P* = 0.9960) dendritic lengths between genotypes. (*APP* V717I pyramidal cells *n* = 9, isogenic pyramidal cells *n* = 14). Each data point represents the mean value from each differentiation where data were collected from three independent inductions from the patient pair. Bar graph represents the mean and SEM, ** *P* < 0.01 * *P* < 0.05.

To investigate the correlation of synaptic and morphological changes, iPSC cells were filled with biocytin (a valuable marker allowing visualization of recorded neurons), fixed post-recording proceeded by electrophysiological assessments; cells labelled with Alexa555-streptavidin were further characterized using inhibitory neuron markers CamKIIa, PV and SST. The cells were recovered using DAB and were reconstructed to reveal the gross dendritic morphology. One example of the recovered iPSC-derived pyramidal cells that were anatomically reconstructed at ×1000 magnification from both *APP* V717I and isogenic co-cultures at 75 and 97 days *in vitro* (DIV) is shown in Fig. 8.

Morphologically, hiPSC-derived pyramidal neurons revealed characteristic pyramidal-shaped somas with one apical and multiple basal dendrites, SST-expressing cells showed oval somata with two primary dendrites from opposing ends and PV-expressing interneurons typically had round somata with multipolar dendrites. However, all hiPSC-derived neurons showed reduced dendritic arbourization and axonal ramification compared to neurons analysed from the *ex vivo* mouse cortex.

An example of the Sholl analysis conducted from DIV 97 iPSC-derived pyramidal cells from each genotype is shown in Fig. 8E, plotting dendritic intersections against distance from the soma. The dendritic complexity of *APP* V717I iPSC-derived pyramidal cells were less extensive, the ramification of the basal dendritic branches (*P* = 0.0083) and apical branch points (*P* = 0.0310) were reduced compared to the isogenic controls as shown in Fig. 8F–H.

## Discussion

Herein, we present a novel co-culture stem cell model designed to more faithfully recapitulate critical brain circuits in the human brain, providing a comprehensive platform for Alzheimer’s disease research. The co-culture system included excitatory neurons, inhibitory neurons, and astrocytes, which were seeded in 12-well plates and cultured for 100 days to achieve neuronal maturation. Initially, excitatory and inhibitory neurons were induced separately during the first 20 days. On Day 35, the two neuronal types were proportionally integrated to establish the co-culture model. Throughout culture progression, neuronal maturation and morphological changes were assessed at various intervals via immunofluorescence imaging. Additionally, electrophysiological recordings assessed fundamental neuronal properties, such as action potential frequency and duration, membrane time constant, and input resistance, at different developmental stages. This model thus provides a robust experimental framework to explore neuronal function and disease mechanisms. Complementary pathology-anchored models using direct neuronal reprogramming in 3D have now recapitulated hallmark late-onset Alzheimer’s disease neuropathology: Aβ deposition, tau tangles, and neuronal loss, in human neurons, underscoring that our platform targets circuit-level function rather than AT(N) biomarkers.^10^

Currently, most iPSC models are single-cell models, primarily focusing on excitatory neurons, which constitute 70% of the human brain.^11^ While the iPSC model developed by Shi *et al*.^8^ introduced a co-culture system of excitatory neurons and astrocytes, inhibitory neurons remain largely neglected. Despite their relatively smaller proportion in the brain, inhibitory neurons are critical for maintaining excitation–inhibition balance, regulating network activity, and preventing hyperexcitability, a major contributor to cognitive decline in neurodegenerative diseases such as Alzheimer’s disease.^12,13^ Our co-culture model incorporates excitatory neurons, inhibitory neurons and astrocytes, better representing the complexity of the human brain. Based on the protocol by Maroof *et al*.,^14^ we used LDN-193189 during the first 12 days of induction to promote neural differentiation of hPSCs into the forebrain spectrum by blocking BMP signalling in conjunction with dual SMAD inhibition. Additionally, XAV939 was used to inhibit Wnt signalling and enhance forebrain induction by preventing activation of posterior signalling pathways. During the subsequent week, we synergistically applied Sonic Hedgehog (SHH) and Purmorphamine to activate the SHH pathway, inducing the expression of ventral forebrain markers essential for inhibitory neuron development. Combining this approach with Shi *et al*.'s excitatory neuron protocol, we seeded excitatory and inhibitory neurons at a 1:1 ratio in the same well on Day 35, resulting in a 87:13 E/I ratio and 1:0.89 neuron/astrocyte ratio by DIV 90, which closely resembles the ratios seen in human cortex.^21,22^ This optimized ratio allowed for successful use of the co-culture model in immunofluorescence and electrophysiological experiments. Interestingly, while the inhibitory-only, excitatory-only and combined inhibitory/excitatory co-culture showed similarities in the aberrant synaptic behaviour shown in Fig. 7, specifically significantly higher spontaneous firing, there were interesting differences observed between the three different co-cultures. These differences include the lack of obvious excitation observed in the SST/PV co-culture and the lack of inhibition in the excitatory/astrocyte co-culture, only the combined co-culture closely resembled synaptic physiology in terms of excitation and inhibition displayed. Furthermore, the combined *APP* V717I showed hyperexcitability compared with their isogenic controls resembling various rodent Alzheimer’s disease models.^15,16^

The electrophysiological properties observed from DIV 75 to DIV 95-100 appear to illustrate the expected increase in electrical maturity with culture duration within each genotype. Interestingly, the membrane and synaptic properties displayed by the *APP* V717I cells compared to isogenic controls corroborated with previous studies that show hyperexcitability and hyperactivity in the Alzheimer’s disease models seen in the rodent brains^3^ but also in iPSC models.^17,18^

The changes in the intrinsic membrane properties were correlated with gross morphology and corroborates with previous studies performed in mouse models of Alzheimer’s disease.^3^ Morphological analyses after 100 days in culture further demonstrated robust dendritic development. Isogenic control pyramidal neurons exhibited extensive dendritic arbourization, indicative of mature morphology. In contrast, neurons expressing *APP* V717I mutation display visibly reduced dendritic complexity, characterized by fewer apical and basal dendritic branches and shorter total dendritic lengths. Such dendritic atrophy mirrors neuropathological features of Alzheimer’s disease, including synaptic and dendritic degeneration, strongly correlated with cognitive impairment.^19^ Consistent with our findings, previous studies also reported impaired neurite outgrowth in human iPSC-derived neurons harbouring the *APP* V717I mutation.^20^ The presence of astrocytes within our co-culture likely promoted neuronal structural maturation, given their known roles in neuritogenesis and synaptogenesis.^23^

Our current assessment of astrocyte reactivity is based primarily on GFAP immunoreactivity, with SOX9 serving as an astrocyte identity marker rather than an activation readout. We therefore view the glial findings as a first pass, and future iterations of this platform will incorporate additional astrocyte reactivity markers (e.g. vimentin or complement components) to resolve reactive substrates in more detail.

PV specifically labels inhibitory neurons characterized by robust dendritic arbours and significantly higher synaptic input density than other inhibitory populations, such as calbindin- or calretinin-positive neurons.^24^ SST marks a distinct subclass of inhibitory GABAergic neurons, enabling detailed analysis of their specific contributions to network function.^25,26^ These markers facilitated comprehensive characterization of inhibitory neuron populations, elucidating their synaptic integration and functional properties within the co-culture system.

Our early-stage immunophenotyping focused on dorsal cortical progenitors (*FOXG1*, *PAX6*, *TBR2*), with some ventral progenitor confirmation using *NKX2.1.* However, as we did not include extensive ventral forebrain marker characterization (e.g. *LHX6*, *DLX1/2*) that are typically used to confirm interneuron lineage commitment, we refrain from inferring inhibitory fate at progenitor stages. Later-stage expression of SST and PV, together with PNN maturation, supports inhibitory interneuron differentiation in our cultures but does not retroactively establish early ventral identity. Accordingly, future work will incorporate more extensive ventral lineage markers and developmental time courses to resolve early specification. As a methods-focused study, we validated the platform in *APP* V717I isogenic control pair to maximize internal validity and minimize inter-donor genetic confounds, with broader validation across additional donor lines planned for future work.

Furthermore, functionally, the SST cells recorded showed an adapting firing pattern, their action potentials were slightly faster than the pyramidal cell recorded, which is consistent with data from rodent studies.^27^ Whether the PV-expressing cells displayed classical fast-spiking properties in our model remains to be fully elucidated, full quantification and characterization of SST-and PV-expressing cells in these co-culture will be investigated in our future studies.

Overall, our co-culture model effectively captures subtle disease-related electrical morphological deficits, underscoring its utility for studying structural and functional neuronal changes in Alzheimer’s disease.

## Conclusions

This study details creating novel *in vitro* human dementia models. The evidence we provide validates this model and demonstrates its potential for a valuable preclinical tool to screen novel targeted therapies and act as a platform for partial replacement for animal experimentation.

## Supplementary Material

fcag135_Supplementary_Data

## Data Availability

The original contributions presented in this study are included in the article and Supplementary Fig. 1. Further inquiries can be directed to the corresponding author(s).

## Code availability

Custom code for patch clamp analysis has been deposited at GitHub: IPSCPAPER2025/IPSC_Methods_Paper_Electrophysiology_analysis: Codes used to analyse data for IPSC methods paper whole-cell electrophysiology recordings.
