# Supplementary material for: Methodology for human-induced pluripotent stem cell–derived excitatory and inhibitory neuron coculture with astrocytes for Alzheimer’s disease modelling
Source: Brain Commun. 2026 May 11;8(3):fcag135. doi: 10.1093/braincomms/fcag135 (PMC13155454; doi:10.1093/braincomms/fcag135)
Supplement: fcag135_Supplementary_Data [file fcag135_supplementary_data.pdf]

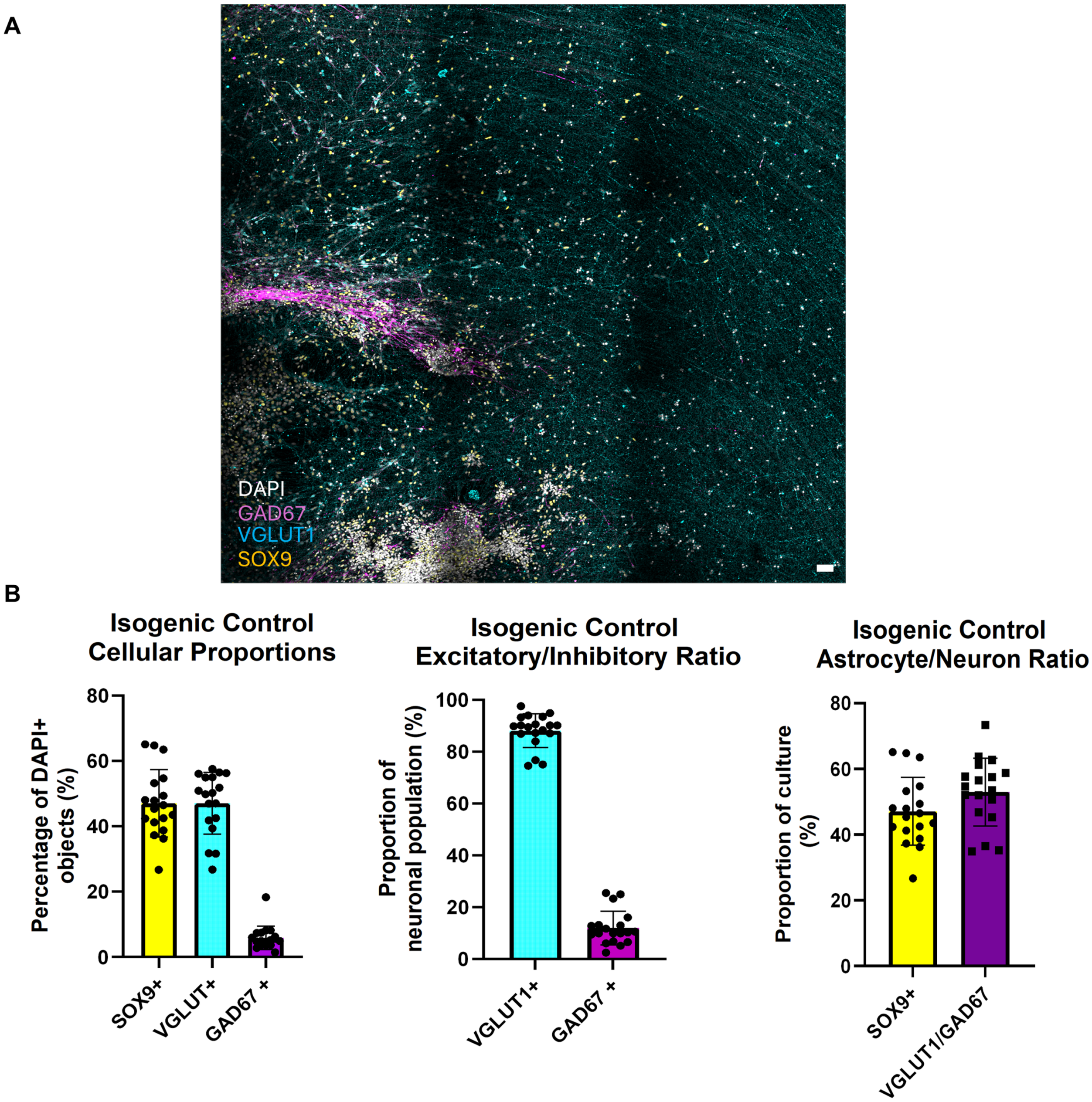

Supplementary Figure 1. Cell type proportions at DIV 90 in the isogenic control line. (A) Representative image displaying the three markers highlighting excitatory neurons (VGLUT1), inhibitory neurons (GAD67) and astrocytes (SOX9). (B) Quantification of marker proportions relative to the whole culture, the neuronal population and the relative proportions of neurons and astrocytes. Each data point corresponds to the proportion of a given cell type from one 3x3 field of view. For each coverslip, 3 x 9 large images (3 x 3) were taken, with three coverslips analysed per induction across three independent inductions. No statistical tests were performed on this data set. Images taken at x20 magnification, scale bar = 50μM.
